# 903 Protein Saver cards: the best alternative for dried blood spot storage at room temperature for HCV RNA

**DOI:** 10.1038/s41598-022-14375-8

**Published:** 2022-06-16

**Authors:** Sonia Arca-Lafuente, Cristina Casanueva-Benítez, Celia Crespo-Bermejo, Violeta Lara-Aguilar, Luz Martín-Carbonero, Ignacio de los Santos, Ricardo Madrid, Verónica Briz

**Affiliations:** 1 Laboratory of Reference and Research on Viral Hepatitis, Centro Nacional de Microbiología, Instituto de Salud Carlos III, Carretera Majadahonda-Pozuelo km 2.2, 28220 Majadahonda, Madrid Spain; 2 BioAssays SL, Parque Científico de Madrid, c/Faraday, 7, Campus de Cantoblanco, 28049 Madrid, Spain; 3 Instituto de Investigación Sanitaria Hospital de la Paz (IdiPAZ), 28046 Madrid, Spain; 4 Servicio de Medicina Interna-Infecciosas, Hospital Universitario de La Princesa, 28006 Madrid, Spain; 5 Department of Genetics, Physiology and Microbiology, Faculty of Biology, Complutense University of Madrid, 28040 Madrid, Spain

**Keywords:** Viral infection, Hepatitis C virus

## Abstract

Hepatitis C virus (HCV) infection remains a global health problem, detected only in the early stages by molecular tests. Molecular tests detect HCV RNA, which is very prone to degradation by ribonucleases, reason why blood samples must be transported and stored at − 20 °C, or even − 70 °C for long-term storage. Flinders Technology Associates (FTA) cards are a useful sampling collecting device for dry blood spot (DBS) storage, especially for low and middle-income countries (LMIC). In this study, we analyzed viral HCV RNA integrity for long-term storage at room temperature compared to − 20 °C using two different types of cards for DBS: FTA Classic and 903 Protein Saver cards. For this purpose, DBS were prepared on these cards using blood or plasma samples from HCV infected patients, and samples were analysed by conventional RT-PCR. Our results showed that 903 Protein Saver cards are the best and cheapest alternative for DBS storage at room temperature. In these conditions, we found that HCV RNA integrity lasted for up to 9 months.

## Introduction

Hepatitis C virus (HCV) is a single-stranded RNA(+) blood-borne virus, that affects an estimated 71 million chronically infected people with all over the world^1^ being one of the main causes for liver cirrhosis related deaths. Up to eight different genotypes have been described so far, showing a differential distribution globally but with the highest prevalence for genotype *1*^2,3^.

Current HCV diagnosis algorithm consists of a serological test followed by a molecular test^4^. Serological tests can detect anti-HCV antibodies or HCV antigens, while molecular tests detect HCV RNA. Anti-HCV antibodies can remain in the organism long time after virus clearance, so they are not a convenient biomarker to determine an active HCV infection. HCV Core antigen can correlate with viremia values, but it has lower sensitivity than molecular tests^5,6^, which makes molecular tests the preferred method to confirm an active HCV infection. Among the different molecular techniques, RT-qPCR is the most used method, although it requires both specialized personnel and expensive equipment. Alternative molecular tests are being developed to overcome RT-qPCR drawbacks, although sampling methods are the main and common limitations. Conventional blood or plasma sample storage has some disadvantages especially for RNA, which is particularly fragile and prone to degradation by ribonucleases^7–9^.

For long-term storage of viral RNA in human plasma, very low temperatures of at least − 70 °C are required. Under these conditions, it has been shown that the integrity of viral RNA from HCV or HIV lasts for up to 9 years whereas at − 20 °C, its stability is notably reduced to only 2 years^10^. To allow an optimal storage of viral RNA at 4 °C, stabilizing buffers containing chaotropic agents have been developed to maintain its integrity for 5 months^11^. On the other hand, some studies have shown that viral RNA remains stable around 1–2 weeks without refrigeration, as it was the case for Ebola virus RNA conserved in EDTA solution^12^ or away from the light on solid surfaces in simulated tropical conditions^13^. Commercial shipment and storage systems for enhanced RNA preservation in solution have been developed. For instance, PAXgene and Tempus systems (from BD and Thermofisher, respectively) contain DNA/RNA stabilizers to transport blood samples at room temperature assuring their stability for up to 5 days^14^. These systems make easier both the storage and handling of samples at analytical laboratories.

Dried blood spot (DBS) is a blood and plasma sampling collecting method firstly used in 1869 for paediatrics screening, to detect phenylketonuria in newborns^15,16^. In those procedures, a filter paper is first soaked with blood drops and then dried at room temperature preserving sample proteins. Later, Flinders Technology Associates (FTA) cards were developed based on this approach. Current FTA cards utilize Whatman FTA technology that contain chemicals that lyse cells, denature proteins and protect nucleic acids from nucleases, UV or oxidation damage, while any potential pathogen present in the sample is completely inactivated^17,18^. For instance, FTA Classic and FTA Elute assure the stability of nucleic acids from whole blood for up to 36 months when stored in a dry atmosphere at − 15 °C^17^. Those characteristics make FTA cards a suitable device for nucleic acid sampling and storage, allowing sample shipment safer without any specific temperature requirement or stabilising agents. Moreover, it has been shown that DBS can be purified and used directly for PCR amplification reducing sample handling^19^. On the other hand, 903 Protein Saver cards have an untreated matrix, and are the best tool to store sample proteins from whole blood and plasma. 903 Protein Saver cards are the FTA alternative technology for conventional DBS^15,16^.

Regarding viral DNA, Hepatitis B virus (HBV) DNA remained detectable between 3 and 5 months stored as DBS at RT conditions ranging from 4 to 37 °C^20^. Regarding viral HCV RNA storage, some authors describe a reduction in RNA detectable levels after 6 days, while others find no decrease after more than 11 months^20^ at room temperature. However, no decrease in HCV RNA integrity was observed upon elution of viral RNA from DBS after 18 months storage at − 20 °C or − 80 °C^21^.

In order to implement HCV screening strategies on hard-to-reach populations, decentralized sample collection is needed and FTA cards, especially FTA Classic cards, are promising candidates since they have been validated as a safe mean of shipment for hazardous samples, and they do not require specific temperature conditions. Moreover, as samples can be quickly purified from FTA cards after several washing steps, they could be handled in low resources settings. Storage time is also crucial to follow virus clearance or viremia relapse.

Our aim was to identify the best FTA card option for DBS to ensure long-term HCV RNA stability and purification yield from either whole blood or plasma samples. Additionally, we aimed to determine whether temperature conditions have any influence in long-term stability of HCV RNA on FTA cards.

## Methods

### FTA cards

HCV RNA stability was analysed in two different FTA Cards: FTA Classic and Protein Saver 903 cards (GE Healthcare, Buckinghamshire, UK). FTA Cards were soaked with HCV-JFH1 culture supernatant, or venous whole blood or plasma samples from HCV patients. Volumes applied were 200 µL for FTA Classic cards or 25 µL for 903 Protein Saver cards. Then, FTA cards were let drying at room temperature for 2 h and FTAs were stored either at room temperature or at − 20 °C. HCV-JFH1 culture supernatant was used as an internal positive control in order to assess temporal differences with HCV RNA stability from blood and plasma patients. HCV-JFH1 culture supernatant (TCID50 = 10^6^ UI/mL) was previously recovered by our group and stored at liquid N_2_ until further use.

### Study population

Six HCV infected adults were recruited from two hospitals in the Community of Madrid (Spain): Hospital Universitario La Princesa and Hospital Universitario La Paz. Samples were collected between June and October 2019. All patients included were chronically infected with HCV (positive PCR and antibodies for HCV) and they had never been treated for HCV infection. Whole blood and plasma samples from HCV infected patients were collected and tested (Table 1).

**Table 1:** Characteristics of the study population.

Sample reference	Sample type	Viral titer (UI/ML)
PT1	Blood and plasma	440,000
PT2	Blood and plasma	7350
PT3	Blood and plasma	1,570,000
PT4	Blood and plasma	193,000
PT5	Blood	56
PT6	Blood and plasma	21,129,963

### Laboratory parameters

Five mL of blood were collected in EDTA tubes. Then, one mL was used to soak two wells of the FTA card [FTA Classic and Protein Saver 903 cards (GE Healthcare, Buckinghamshire, UK)], and four mL were used to separate plasma from blood by a density gradient.

Plasma HCV RNA viral load was measured by COBAS TaqMan HCV Test (Roche Diagnostic Systems Inc), with a detection limit of 18 RNA copies/mL. The STROME-ID checklist was used to strength the design and conduct the study^22^. Sample viral load and the samples are indicated in Table 1.

### RNA purification from FTA cards

Cards were subjected to thaw/freeze cycles at each time point along the study. RNA was purified using Whatman Purification Reagent (GE Healthcare, Buckinghamshire, UK), following manufacturer’s instructions. Briefly, a 3 mm hole puncher was used to cut one disc from each FTA card and placed in a clean 1.5 mL Eppendorf tube. The 3 mm Ø FTA-disc was washed three times with Whatman Purification Reagent and washed twice with TE buffer (pH 8), and incubated for 10 min at RT. Finally, the discs were allowed to dry at 56 °C for 30 min.

## HCV-RNA amplification and detection

HCV-RNA was amplified using a nested RT-PCR protocol using purified viral RNA extracted from plasma samples or directly on washed FTA cards discs.

### RT-PCR

cDNA synthesis was performed by RT-PCR using the One-Step AffinityScript Multiple Temperature cDNA Synthesis Kit (Agilent Technologies Inc., La Jolla, CA, USA), following manufacturer’s instructions. Briefly, RT-PCR was performed in 50 μL reaction mixture containing: 10 μL of 5X Herculase II buffer, 1 μL of 40 mM dNTPs, 1 μL of 10 μM forward primer, 1 μL of 10 μM reverse primer, 0,5 μL DMSO, 1 μL of a 1:20 dilution of Affinity Script RT (Agilent Technologies Inc., La Jolla, CA, USA), 1 μL Herculase II Fusion DNA Polymerase (Agilent Technologies Inc., La Jolla, CA, USA), 100 ng template and MilliQ water up to 50 μL. For those RT-PCR assays directly on DBS discs, one 3 mm Ø washed disc was added to the mix instead of purified template. The RT-PCR protocol started with the synthesis of cDNA by retro-transcription at 48 °C for 30 min, followed by DNA amplification at 95 °C for 1 min, 40 cycles of 95 °C for 30 s, 55 °C for 30 s and 68 °C for 10 min, and a final extension at 68 °C for 4 min in Mastercycler Nexus GX2 thermal cycler (Eppendorf AG, Hamburg, Germany). Primers used were: forward primer 5′-CTGTCTTCACGCRGAAAGCG-3′ and reverse primer 5′-GCTAGCCGTGACTAGGGCTAAG-3′.

### Nested PCR

Nested PCR was performed to check HCV DNA integrity. We carried out a PCR-amplification of a 500 bp fragment, corresponding to its 5′ Core region. DNA synthesis was performed using Maxime PCR PreMix (i-Taq) (Intron Biotechnologies, Kyungki-Do, Korea) by adding 20 μL reaction mixture containing: 1 μL of 10 μM forward primer, 1 μL of 10 μM reverse primer, 10 ng of HCV DNA, and MilliQ water up to 20 μL. The PCR conditions included an initial denaturation step at 95 °C for 1 min, followed by 35 cycles at 5 °C for 30 s, 55 °C for 30 s and 72 °C for 30 s, using a Mastercycler Nexus GX2 thermal cycler (Eppendorf AG, Hamburg, Germany). Two different primers sets were used: (1) Core region: forward primer 5′-GTATGAGTGTCGTACAGCCTC-3′ and reverse primer 5′-TCATTCCCATATAGGGGCCAG-3′; (2) NS5B region^23^: forward primer 5′-TATGAYACCCGCTGYTTTGACTC-3′ and reverse primer 5′-GCNGARTAYCTVGTCATAGCCTC-3′. PCR products were visualized by electrophoresis in 1% agarose-TAE gels.

### DNA purification

PCR products were purified using Wizard SV Gel and PCR Clean-Up System (Promega, Madison, WI). Purified DNA was eluted in 50 µL of MilliQ water.

### DNA analysis

Two different analyses were performed: nucleic acid quantification, using the UV–Vis spectrophotometer NanoDrop 2000 (Thermo Fisher Scientific, USA) to measure absorbance at 260 nm; and DNA electrophoresis in 1% agarose-TAE gels for 10–20 min at 100 V (Mupid-One Electrophoresis System). DNA was visualized under UV light by incubation with RedSafe dye (Intron Biotechnologies, Kyungki-Do, Korea). Gel band Mean Florescence Intensity (MFI) was determined using ImageJ software^24^. MFI threshold was set at 400 a.u., since confirmation by automated sequencing was not obtained with those PCR fragments showing a MFI value lower than 400 a.u.

### DNA sequencing

PCR products were purified and checked by Sanger sequencing using the following primers: (1) Core region: forward primer 5′-GTATGAGTGTCGTACAGCCTC-3′ and reverse primer 5′-TCATTCCCATATAGGGGCCAG-3′; (2) NS5B region^23^: forward primer 5′-TATGAYACCCGCTGYTTTGACTC-3′ and reverse primer 5′-GCNGARTAYCTVGTCATAGCCTC-3′ (Macrogen INC, Madrid). Sequence analysis was performed using SnapGene software (from Insightful Science; available at snapgene.com) followed by BLASTn alignment at NCBI database.

### Phylogenetic analysis

To further confirm sequence results and the corresponding HCV genotype from samples, phylogenetic inference studies were conducted for HCV RNA sequences using the Molecular Evolutionary Genetics Analysis (MEGA) version 10.2.2. HCV RNA sequences were obtained by nested RT-PCR amplification of total viral RNA extracted from plasma samples. Reference HCV RNA sequences employed in this study included the following HCV subtypes: *1a* (AF009606.1), *1b* (D90208.1), *2a* (AB047639.1, *JFH1*; JQ745652.1, *J8CC*), *2b* (AB559564.1), *3a* (D17763.1), *3b* (D49374.1), *4a* (Y11604.1), *4b* (FJ462435.1), and *4c* (FJ462436.1).

### Ethical statement

The study protocol was approved by the Research Ethics Committees in all institutions involved [Instituto de Salud Carlos III (CEI PI 25_2018-v2), Hospital La Paz (PI-3691), Hospital Universitario La Princesa (3917)]. This study was developed in accordance to the Helsinki Declaration. An informed consent was obtained from each subject, and confidentiality and privacy were assured.

## Results

### Effects of long-term storage on viral RNA integrity

**Figure 1:**
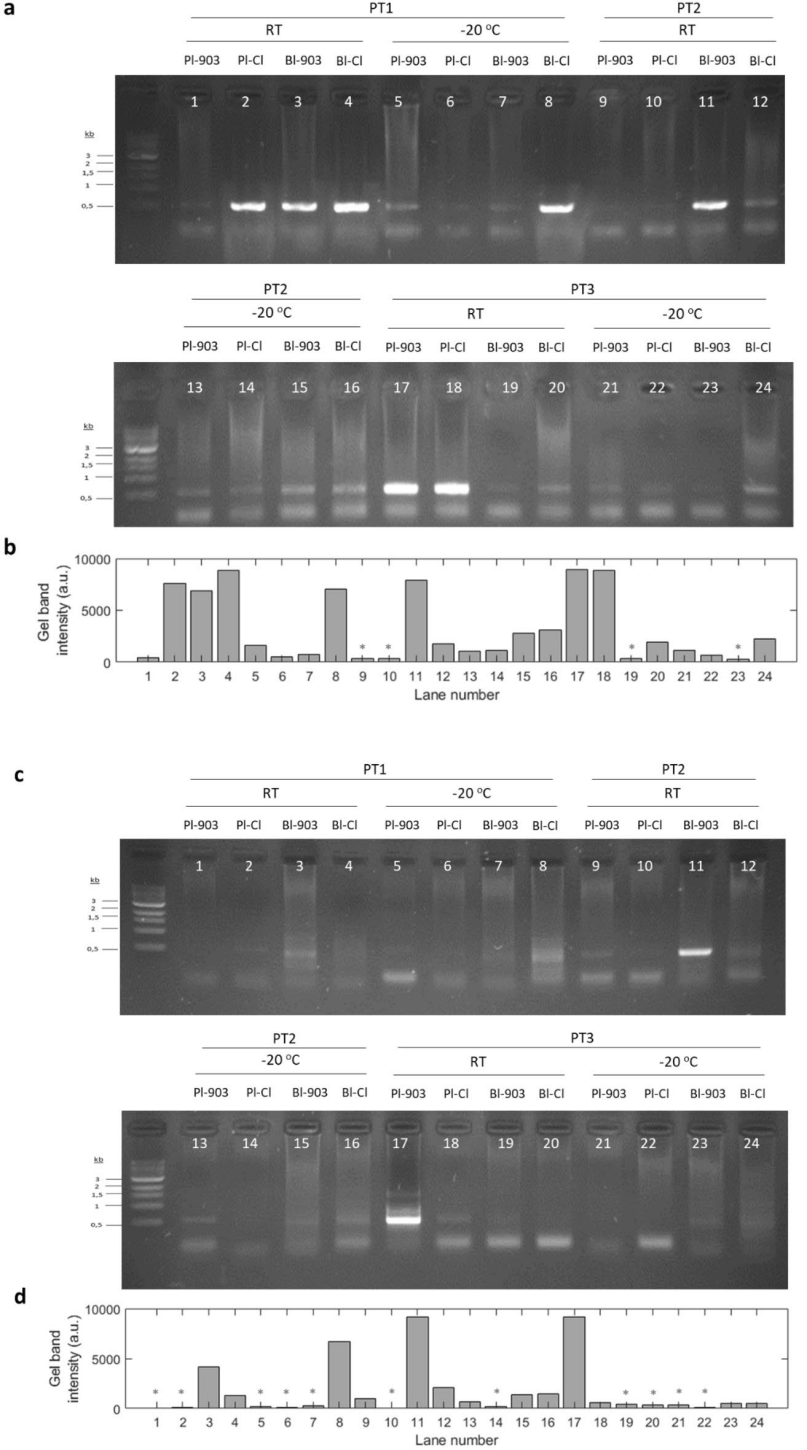
Representative results of PCR amplification in samples PT1, PT2 and PT3 at the different storage and temperature conditions analysed. In upper panels, images correspond to the migration pattern of the PCR products (**a**), and histograms represent their measured MFI (**b**) after 255 days of storage. In lower panels, similarly, images correspond to the migration pattern of the PCR products (**c**), and histograms represent their measured MFI (**d**) after 360 days of storage; Pl-903: plasma sample stored in 903 Protein Saver Card; Pl-Cl: plasma sample stored in FTA Classic Card; Bl-903: blood sample stored in 903 Protein Saver Card; Bl-Cl: blood sample stored in FTA Classic Card. (*) Negative samples.

We first evaluated the integrity of HCV RNA along time in different FTA cards and temperature conditions by RT-PCR assays (Fig. 1). PCR fragments were purified and sequenced to confirm that PCR amplification was specific (Fig. 1S, Supplementary Material). As shown in Fig. 1, the number of positive detections of the HCV-Core from DBS (whole blood or plasma from PT1, PT2 y PT3 samples) was twofold higher at 255 days than at 360 days of storage. In fact, we could detect the viral RNA in 83% (n = 20/24) of the cases at 255 days, while only in 54% (n = 13/24) at 360 days, indicating that the integrity of viral RNA was, at least, optimal after 9 months of storage on DBS.

### Effects of temperature conditions on viral RNA integrity

**Figure 2:**
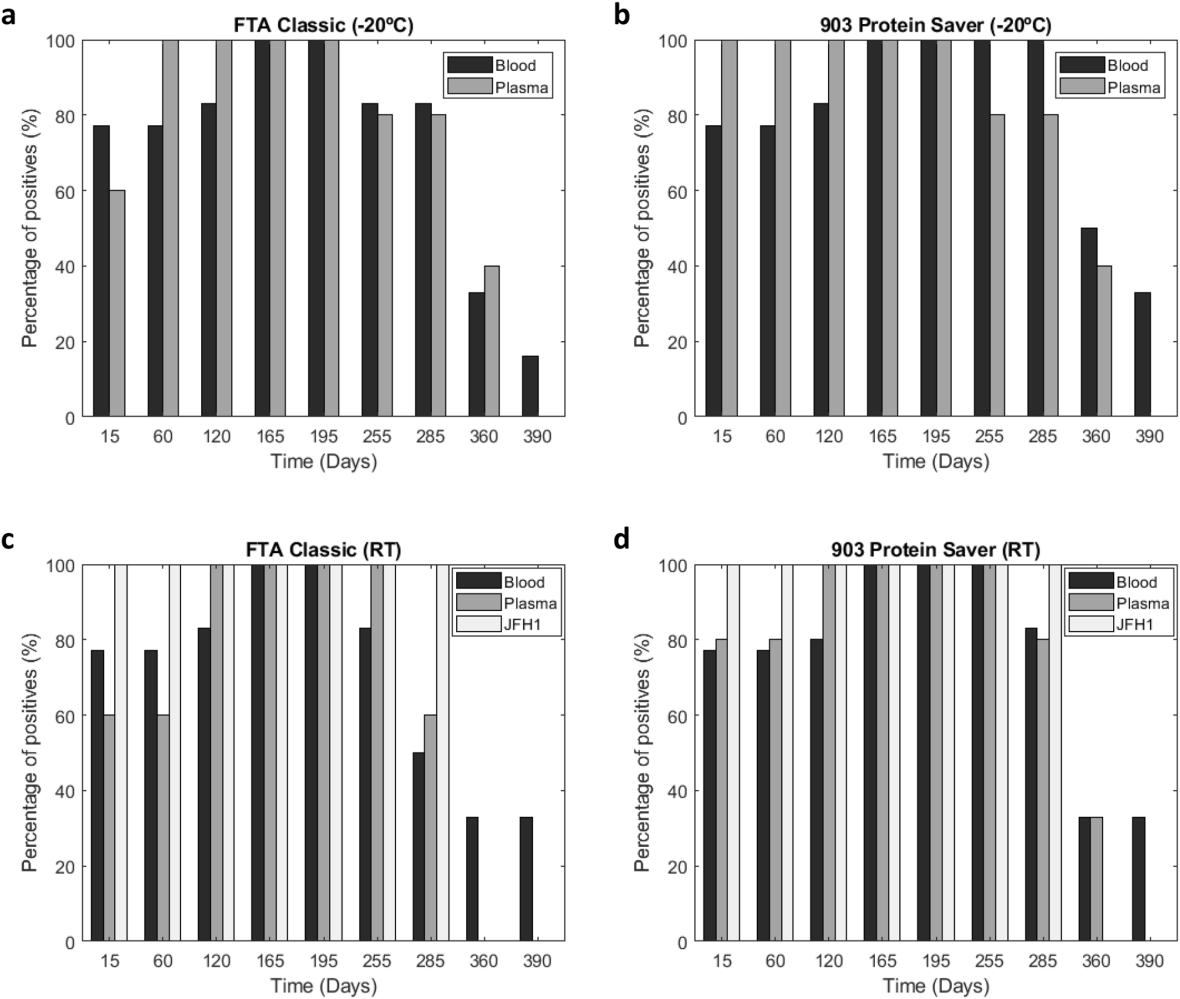
HCV RNA detection in DBS by RT-PCR followed by nested PCR amplification at the different time points analysed, for blood and plasma samples stored in FTA Classic cards (**a**) or 903 Protein Saver cards (**b**) at − 20 °C, and in FTA Classic cards (**c**) or 903 Protein Saver cards (**d**) at room temperature (RT). Histograms represent the % of positive samples at the different time points analysed.

As shown in Fig. 2, the integrity of HCV RNA in FTA Classic cards was slightly higher in samples stored at − 20 °C compared to RT conditions. At least 81% (n = 9/11) of samples stored at − 20 °C tested positive after nested RT-PCR amplification at day 285, while HCV RNA was detectable in less than 54% (n = 6/11) of samples stored at RT (Fig. 2a,c). Of note, HCV RNA stability in 903 Protein Saver cards was not different in cards kept at room temperature (RT) compared to − 20 °C conditions (Fig. 2b,d). At day 360, HCV RNA stability in FTA Classic cards was up to 33% (n = 2/6) in blood samples stored at either RT or − 20 °C, while 40% (n = 2/5) of plasma samples tested positive when stored at − 20 °C. Conversely, and HCV RNA was undetectable when stored at RT at this time point. At day 390, 33% (n = 2/6) blood samples tested positive, but only 16% (n = 1/5) plasma samples did.

### Effect of storage cards on RNA integrity: FTA classic vs 903 Protein Saver cards

**Figure 3:**
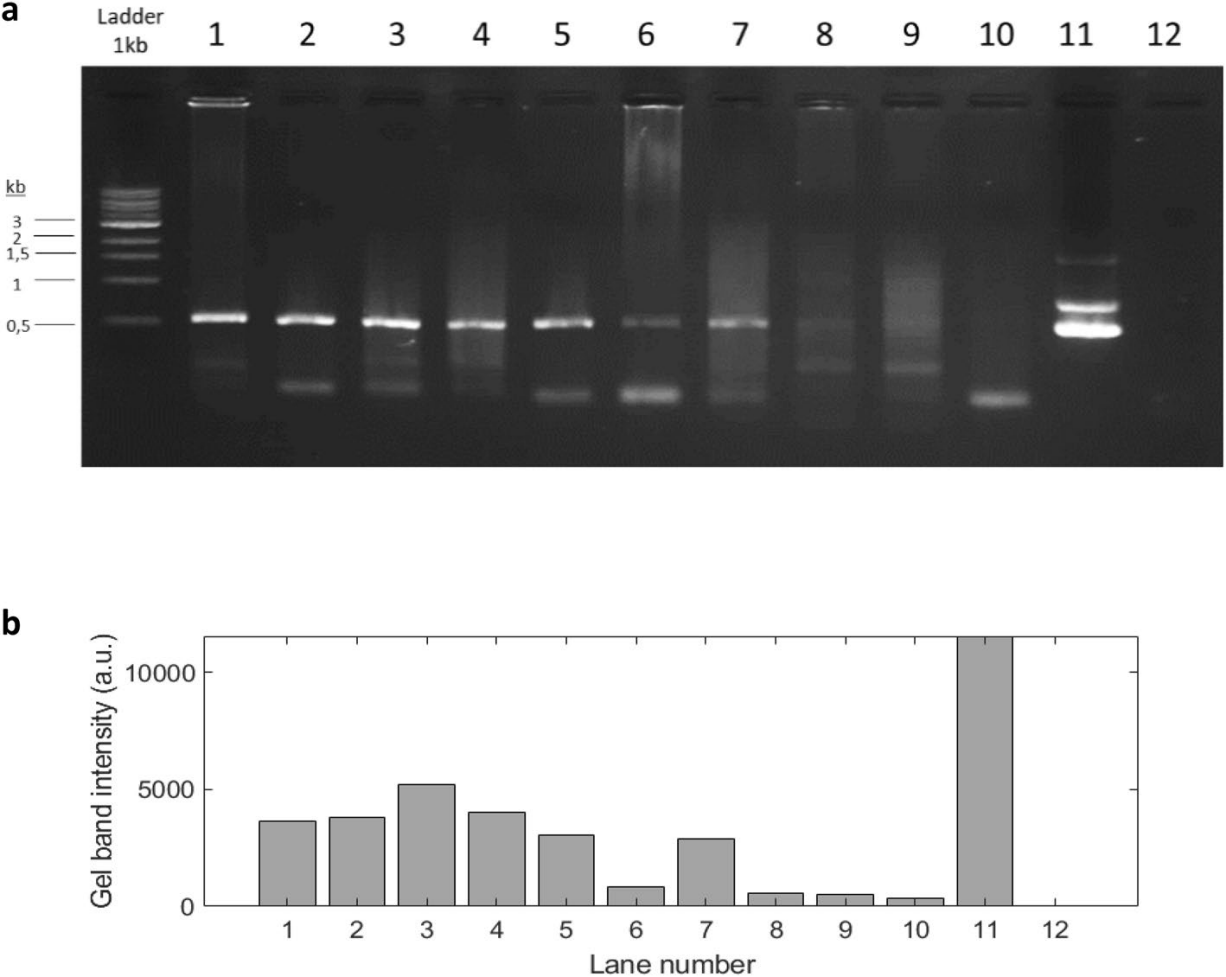
Representative PCR amplification profile for HCV-core region overtime using blood samples of patient PT1 stored in 903 Protein Saver cards at room temperature. (**a**) Agarose gel electrophoresis of the PCR products; (**b**) Histograms represent a single MFI analysis of PCR products over time. Lane 1–10: amplicons obtained at 15, 60, 90, 120, 165, 195, 255, 285, 360, 390 days of analysis, respectively. Lane 11: positive control pUC-JFH1 plasmid. Lane 12: non template control.

As shown in Figs. 2 and 3, a slightly higher HCV RNA stability was observed in samples stored in 903 Protein Saver cards (Fig. 2b,d) compared to FTA Classic cards (Fig. 2a,c) in blood and plasma samples stored at RT or − 20 °C conditions.

Therefore, we focused on RT storage to analyze the effect of sample storage cards, since it is the preferred storage temperature for point-of-care (POC) diagnostics.

At day 255 of analysis, integrity of HCV RNA was very high all along the samples stored at RT in 903 Protein Saver cards. In fact, HCV RNA was always detected in blood and plasma samples stored on these cards, whereas HCV RNA stability was up to 83% (n = 5/6) in blood samples and 100% in plasma samples stored on FTA Classic cards (Fig. 2d).

At day 285 of analysis, at least 81% (n = 9/11) of samples stored in 903 Protein Saver cards, remained positive. However, at day 360, only 36% (n = 4/11) of samples tested positive and it dropped to just one blood sample at day 390 (Fig. 2d).

Positive detection of HCV RNA from JFH1 cell cultures lasted 285 days in both sample storage cards, when stored at RT (Fig. 2d). The analysis performed in samples stored in FTA Classic cards at RT showed that at day 255, 83% (n = 5/6) of blood samples tested positive and 100% of plasma samples or supernatants of JFH1 cell culture. However, at day 285 the percentage of positive samples stored in FTA Classic cards decreased to 50% (n = 3/6) and 60% (n = 3/5) in blood and plasma samples, respectively. At days 360 and 390, only 33% (n = 2/6) of blood samples tested positive, and both plasma and supernatants of JFH1 cell culture tested negative (Fig. 2c).

### Time course analysis on RNA integrity in DBS

**Figure 4:**
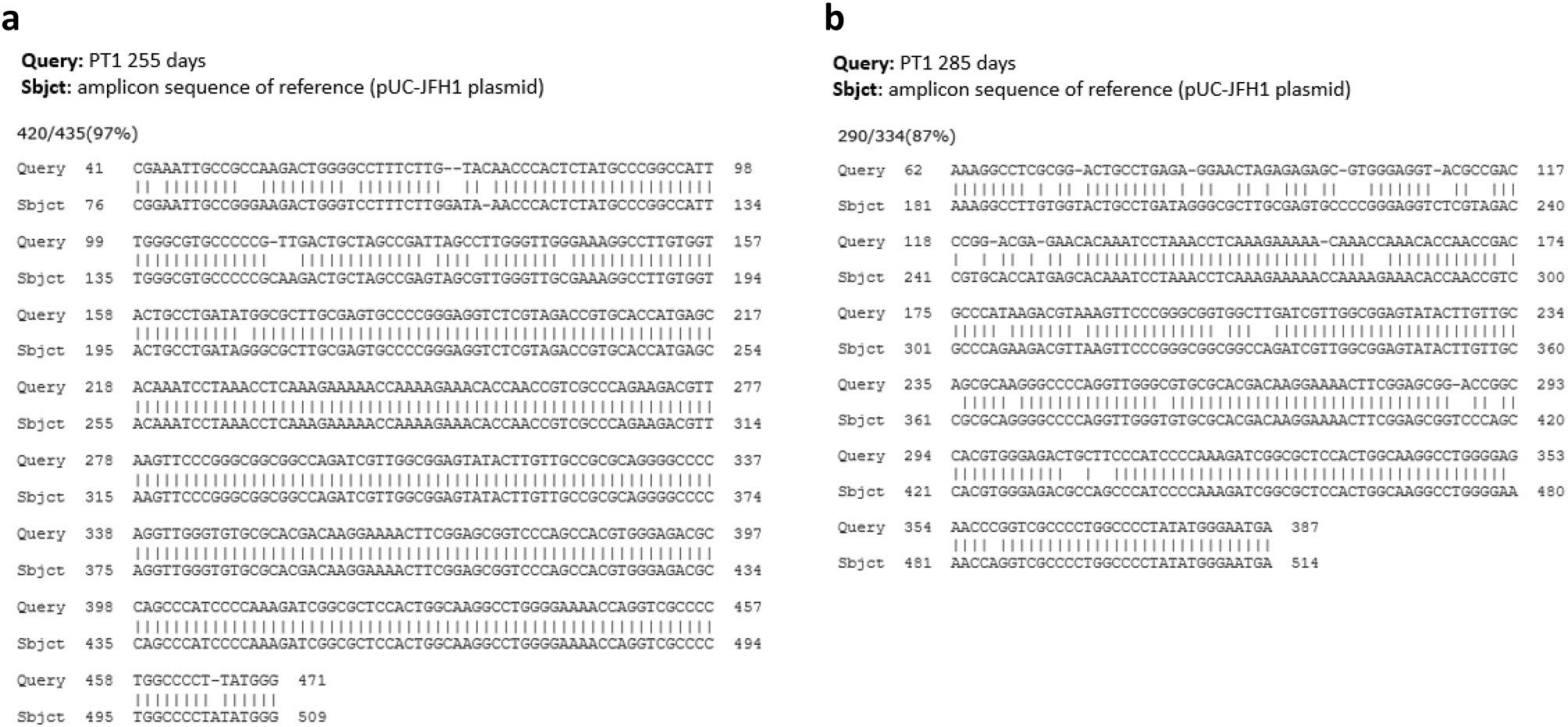
Sequence alignment of PCR fragments from a representative sample (PT1) at 255 days (**a**) or 285 days (**b**) of storage in 903 Protein Saver cards at RT conditions.

To check the HCV RNA integrity overtime in 903 Protein Saver cards at RT conditions, PCR products obtained at the time points 255, 285, 360 and 390 days were purified and sequenced. As shown in Fig. 3, HCV RNA integrity on these cards was noticeable over more than 12 months. In fact, even a slight PCR amplification can still be observed at 285 and 360 days of analysis. No amplification was observed at 390 days. Sequence analysis showed that whole blood samples stored in 903 Protein Saver cards maintained RNA integrity after 255 days of storage showing a 97% sequence identity (Fig. 4a). This value decreased to 87% identity after 285 days (Fig. 4b). No nucleotide sequence was obtained using PCR products obtained at any further time points from 360 days of sample card storage.

Overall, these results clearly suggest that 903 Protein Saver cards are the best device for HCV RNA storage from whole blood or plasma samples even at RT conditions.

### Phylogenetic classification of HCV subtypes

**Figure 5:**
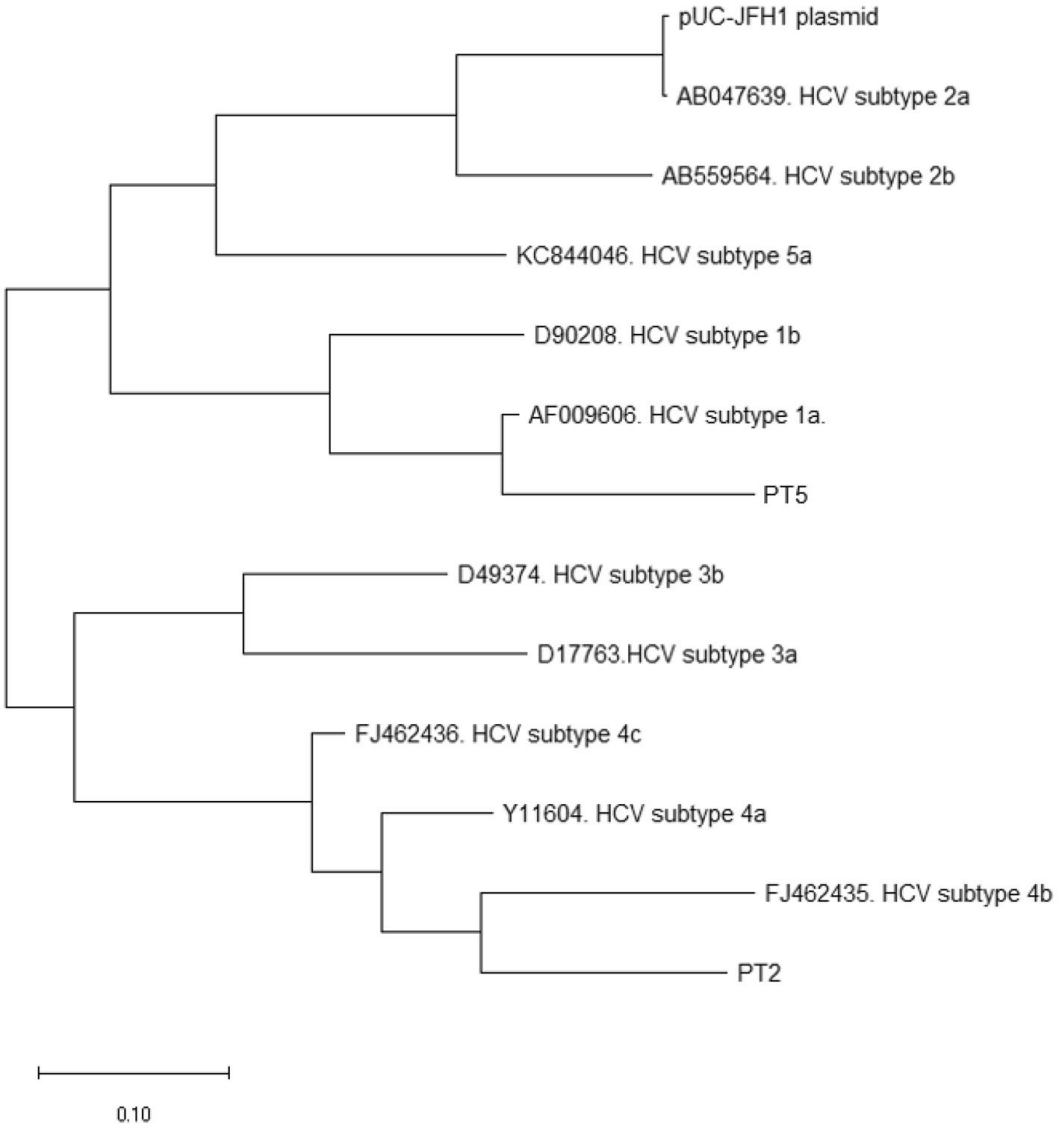
Phylogenetic tree of NS5B region from HCV reference sequences, pUC-JFH1 plasmid, and sequence results from PCR of samples PT2 and PT5.

In order to further validate the DBS in FTA cards as a useful system to study the genetic heterogeneity of HCV virus after long-term storage of its RNA in DBS, we constructed a phylogenetic tree based on the analysis of the NS5B sequence obtained from plasma samples, HCV reference sequences and viral sequence from pUC-JFH1 plasmid. The results showed that HCV subtypes are correctly grouped based on NS5B region (Fig. 5). In fact, the phylogeny results showed that patient PT2 corresponds with HCV subtype *4b*, and patient PT5 corresponded with subtype *1a*.

## Discussion

In this study, we evaluated HCV RNA stability stored as DBS of whole blood or plasma samples using two distinct card devices subjected at different storage conditions. In our hands, we observed a weaker RT-PCR sensitivity during the first time points tested, although it increased after 120 days, possibly due to the optimizations carried out in the purification protocol of FTA cards. As well, it is noteworthy that cards were exposed to thaw/freeze cycles along the study that could have influenced its stability.

Despite these facts, we were able to determine that the 903 Protein Saver cards ensure HCV RNA stability and integrity for longer periods of at least 9 months. Although, slight differences were observed on HCV RNA stability in cards stored at − 20 °C, since in the 30% of samples analysed so far it lasted up to 10 months. Moreover, even if a longer RNA stability was initially expected for samples stored at − 20 °C^21,25^, we could not determine any temperature-dependent effect on HCV RNA stability using 903 Protein Saver cards.

Although FTA Classic cards are supposed to preserve nucleic acids for longer periods of time than untreated cards like 903 Protein Saver, our analysis showed no significant differences between them. Furthermore, we have determined that viral RNA can be preserved at room temperature for 9 months without suffering important degradation using 903 Protein Saver cards. Our results agree with other studies also showing that these cards are suitable for longer nucleic acid storage^26^. In this line, our results are in agreement with those studies done by Keeler et al.^27^.

Regarding sample type, our results showed higher detection rates in whole blood than in plasma samples. During plasma separation, an HCV RNA fraction can be lost due to virus adherence to red blood cells or even by formation of virus-immunoglobulin complexes. Intracellular RNA from those pelleted complexes will be lost in the plasma fraction, and then reduce viral load in plasma compared to whole blood, as has been described by Schmidt et al.^28^.

We have determined that whole blood can be an adequate source of HCV RNA, and that no viral load is lost compared to the mostly preferred plasma sample. Then, 903 Protein Saver cards are an ideal sample device, less expensive than similar developed sampling methods such as Cobas plasma separation card (PSC), developed for HIV-1 quantitation^29,30^. To determine if 903 Protein Saver cards could substitute PSC cards, more studies analysing 903 Protein Saver cards stability in high humidity conditions are required.

Considering the above results, we suggest the 903 Protein Saver cards as the best and the most cost-effective storage system for DBS, since blood samples remained stable further time than FTA Classic cards even at RT conditions. This feature is instrumental for low-resource settings since no additional sample processing is needed for whole blood sample collection, storage, or shipment. Additionally, long-term RNA stability is crucial for patients follow-up.

Long-term storage of baseline samples remains essential to assess viremia relapse after treatment by RT-PCR and sequencing of paired baseline and post-treatment samples. Sequencing and phylogenetic analysis allows us to discriminate between reinfection (different virus than baseline) and treatment failure (same virus). This is especially important in settings where reinfection is common, such as among people who inject drugs.

However, 903 Protein Saver cards have an untreated matrix which does not guarantee virus inactivation. In fact, a recent study showed that purified Newcastle disease virus remained infectious in DBS after 24 h, in contrast to FTA cards^31^. Further studies analysing viral stability in patient samples would be necessary to check 903 Protein Saver cards safety levels for short and long-term storage of blood samples.

Earlier studies using other viruses have been able to detect viral DNA from African Swine fever virus (ASFV) or viral RNA from Peste des Petits Rumiants (PPR) in FTA cards soaked with whole blood of infected animal stored as DBS for 9 months at 22, 32 or 37 °C^32^. In contrast, other studies stablish a maximum FTA card storage at room temperature in 30 days for the detection of Avian Influenza virus RNA^27^. Different detection methods used in these studies, as real-time RT-PCR versus conventional RT-PCR, as well as other procedure differences such as sample volume or RNA extraction method yields, could explain the reported deviations. Due to our main objective along this study, which was to evaluate FTA Classic and 903 Protein Saver cards as an alternative sampling method for HCV infected patients, our principal purpose was to determine if HCV RNA was detected or not after storage in DBS, and for how long it remained detectable by RT-PCR, the methodology use in low and middle-income countries. Conventional RT-PCR is a convenient method for this purpose since no RNA quantitation was needed. However, to certainty conclude that DBS cards are suitable for current HCV diagnostic methods, FTA cards should be validated with established routine qRT-PCR methods.

Due to the presumed RNA weakness, it should better be stored at − 80 °C. However, our main purpose was to analyse RNA integrity as DBS for its implementation in LMIC, with practically no access to − 80 °C storage or shipment conditions. Thus, our results figure out that 903 Protein Saver cards, or even FTA Classic cards, could be a suitable method for long-term HCV RNA storage at room temperature.

In summary, this study shows that non-purified HCV RNA from blood samples can be stored at room temperature in 903 Protein Saver cards for at least 9 months without affecting RNA integrity, which makes 903 Protein Saver cards a recommendable sampling method for HCV diagnosis in LMIC countries.

## Supplementary Information

Supplementary Figure S1.

## Data Availability

Data will be available upon request to Dr. Verónica Briz (veronica.briz@isciii.es).
