# Supplementary material for: 903 Protein Saver cards: the best alternative for dried blood spot storage at room temperature for HCV RNA
Source: Sci Rep. 2022 Jun 16;12:10124. doi: 10.1038/s41598-022-14375-8 (PMC9203708; doi:10.1038/s41598-022-14375-8)
Supplement: Supplementary file 1 — Supplementary Figure S1. [file 41598_2022_14375_MOESM1_ESM.docx]

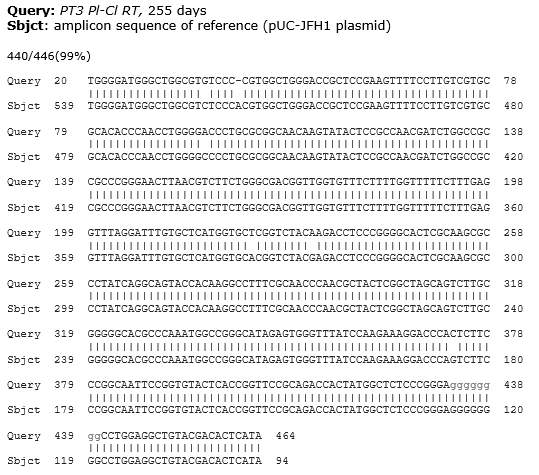


**Figure 1S**. Representative sequence analysis of a PCR fragment from patient PT3 (plasma sample stored in FTA Classic Card^®^ at RT) at 255 days of storage.
